# Dynamic MAML with Efficient Multi-Scale Attention for Cross-Load Few-Shot Bearing Fault Diagnosis

**DOI:** 10.3390/e27101063

**Published:** 2025-10-14

**Authors:** Qinglei Zhang, Yifan Zhang, Jiyun Qin, Jianguo Duan, Ying Zhou

**Affiliations:** China Institute of FTZ Supply Chain, Shanghai Maritime University, Shanghai 201306, China; qlzhang@shmtu.edu.cn (Q.Z.); 202330210078@stu.shmtu.edu.cn (Y.Z.); jyqin@shmtu.edu.cn (J.Q.); jgduan@shmtu.edu.cn (J.D.)

**Keywords:** few-shot learning, efficient multi-scale attention, adaptive meta-learning, cross-domain fault diagnosis

## Abstract

Accurate bearing fault diagnosis under various operational conditions presents significant challenges, mainly due to the limited availability of labeled data and the domain mismatches across different operating environments. In this study, an adaptive meta-learning framework (AdaMETA) is proposed, which combines dynamic task-aware model-independent meta-learning (DT-MAML) with efficient multi-scale attention (EMA) modules to enhance the model’s ability to generalize and improve diagnostic performance in small-sample bearing fault diagnosis across different load scenarios. Specifically, a hierarchical encoder equipped with C-EMA is introduced to effectively capture multi-scale fault features from vibration signals, greatly improving feature extraction under constrained data conditions. Furthermore, DT-MAML dynamically adjusts the inner-loop learning rate based on task complexity, promoting efficient adaptation to diverse tasks and mitigating domain bias. Comprehensive experimental evaluations on the CWRU bearing dataset, conducted under carefully designed cross-domain scenarios, demonstrate that AdaMETA achieves superior accuracy (up to 99.26%) and robustness compared to traditional meta-learning and classical diagnostic methods. Additional ablation studies and noise interference experiments further validate the substantial contribution of the EMA module and the dynamic learning rate components.

## 1. Introduction

With the rapid growth of modern industries and manufacturing, rotating machinery and equipment are widely utilized in key sectors such as aerospace, automotive production, and energy generation. Bearings, as essential components of rotating machinery and equipment, play a crucial role in ensuring the safety, reliability, and efficiency of equipment operations [1]. Failing to detect bearing faults promptly can lead to equipment malfunction, causing substantial economic losses or even casualties. Therefore, the timely and accurate detection of bearing faults is of great practical importance [2,3].

Traditional signal processing and analysis techniques have been extensively applied in the diagnosis of faults in rotating machinery; however, these methods often struggle to meet the increasingly complex requirements of modern equipment, particularly in terms of accuracy and efficiency. They are especially ineffective in varying operational conditions and noisy environments. Early machine learning models, such as K-Nearest Neighbors (KNNs) [4], Artificial Neural Networks (ANNs) [5], and Support Vector Machines (SVMs) [6], when combined with feature extraction techniques in signal processing, have shown improvements and have enhanced fault identification performance [7]. However, these approaches rely heavily on manual feature engineering, which can result in information loss and a degradation of classification performance if poor features are selected. With advancements in chip technology, deeper neural network models can now be efficiently trained. Deep learning (DL) models have the capability to automatically extract features directly from raw signals, eliminating the need for manual preprocessing, which results in greater robustness and improved performance [8]. These models have achieved notable success in bearing fault diagnosis. Specifically, deep learning approaches such as Convolutional Neural Networks (CNNs) [9,10], Recurrent Neural Networks (RNNs) [11], and Transformers [12,13] have been widely utilized in bearing fault diagnosis due to their robust automatic feature extraction abilities, which significantly enhance diagnostic accuracy, particularly in complex operational conditions.

However, there are two major bottlenecks in these approaches:

(a) Sample-dependent problem: Existing methods require a large number of labeled fault samples to achieve high accuracy [14]. In real industrial scenarios, the scarcity of early fault samples, variable working conditions, and the high cost of labeling create challenges in meeting data requirements, as data distribution varies significantly [15].
(b) Poor adaptability to dynamic working conditions: In variable-speed and high-noise environments, traditional feature extraction methods struggle to capture weak fault features. Moreover, in practical applications, equipment is typically shut down immediately once a fault occurs, preventing the collection of sufficient samples for model training [16]. Therefore, more effective methods are needed for diagnosing bearing faults in real-world industries, especially for bearings operating under diverse conditions with limited data.

To tackle the challenge of limited data, Few-shot Learning (FSL) techniques have attracted growing interest in recent years. Luo et al. (2024) introduced an Elastic Prototypical Network that improves transfer diagnosis robustness under unstable rotational speeds [17]; Jiang et al. (2024) designed a Recursive Prototypical Network with Coordinate Attention to enhance separability in few-shot cross-condition scenarios [18]; Lin et al. (2025) proposed a Prototype Matching-based meta-learning model tailored for constrained-data diagnosis [19]; Li et al. (2024) developed Learn-Then-Adapt, a test-time adaptation scheme enabling on-the-fly cross-domain adaptation without target labels [20]; Cui et al. (2024) presented a Dictionary Domain Adaptation Transformer to alleviate cross-machine distribution shift by dictionary-level alignment [21]; Yan et al. (2023) built LiConvFormer, a lightweight separable-multi-scale convolution plus broadcast self-attention framework for efficient deployment [22]; Liu and Peng (2025) proposed a semi-supervised meta-learning approach with simplifying graph convolution for variable-condition few-shot diagnosis [23]; Zhu et al. (2024) formulated a cloud–edge test-time adaptation pipeline with customized contrastive learning for online machinery diagnosis [24]; Li et al. (2025) introduced a Multi-Variable Transformer-based meta-learning architecture that couples Transformer encoders with MAML for multivariate time series [25]; and Xiao et al. (2025) provided a comprehensive survey on domain generalization for rotating machinery, consolidating settings, benchmarks, and open issues [26].

Meta-learning is a central strategy within FSL. It has been demonstrated that this facilitates a rapid and effective adaptation to new tasks, with minimal data, by means of learning efficient learning strategies. This method has demonstrated considerable benefits, especially in the context of bearing fault detection [27]. Meta-learning approaches are generally divided into three types: optimization-based methods, model-based methods, and metric-based methods [28]. Among these, optimization-based approaches are designed to offer a globally shared initialization for all meta-tasks [29], helping the model to rapidly achieve superior classification accuracy with only minor parameter adjustments and a small amount of data. Traditional optimization-based methods, such as model-independent meta-learning (MAML) [30], substantially improve the model’s ability to quickly adapt to new tasks by setting shared initial weights during the meta-training phase [31,32].

In recent times, notable advancements have been made in applying meta-learning to diagnosing faults in rotating machinery. For example, Wang and Liu (2025) [33] proposed a multi-scale meta-learning network (MS-MLN), which integrates a multi-scale feature encoder with a metric embedding strategy. This network effectively combines data from multiple scales without the need for manual feature extraction, leading to quick generalization at the task level. Lin et al. (2023) [34] introduced the GMAML algorithm, which is specifically tailored to solve the issue of small-sample cross-domain bearing fault detection problems driven by diverse signals (such as acceleration/acoustics). The development of the channel interaction feature encoder (MK-ECA) was based on multi-core efficient channel attention and included a weight guidance factor (WGF) in the inner optimization of MAML, which adaptively tunes the training strategy and substantially enhances cross-domain generalization. Su et al. (2022) [35] proposed the DRHRML method, which integrates Maximum Mean Discrepancy (MMD) constraints via the Improved Sparse Denoising Autoencoder (ISDAE) for data reconstruction. This approach reduces noise and maintains distributional consistency, achieving fast adaptation to small sample sizes and cross-task generalization through MAML-based recursive meta-learning (RML), leading to significant test accuracy improvements under various working conditions. Dong et al. (2025) [36] introduced MTFL, aimed at small-sample cross-domain bearing fault diagnosis under diverse operating conditions. In their approach, 1D vibration signals are converted to 2D images (STI and MSMY branches), features are extracted using multi-source pre-trained ResNet18, and multi-source, two-branch features are selected and fused using SRF. The domain gap is narrowed through Domain Adaptation (DA) with a Learning Linear Adaptor, and the final classification is performed with a prototype network.

Although the above methods perform well across different tasks and operating conditions, the traditional MAML algorithm still has certain limitations, particularly when applied to cross-domain tasks. the inner-loop learning rate in MAML is fixed and does not adjust dynamically with the complexity of the task or changes in the data. This rigid learning rate strategy limits the model’s ability to adapt and generalize in more intricate and fluctuating task scenarios. Moreover, the varying complexity of bearing equipment operating conditions introduces challenges for fault diagnosis, especially in relation to cross-domain generalization. There are often notable differences in data distribution between the source and target domains, which makes it difficult to apply models trained on the source domain directly to the target domain, thus affecting diagnostic performance [37,38].

To address these challenges, this paper presents an adaptive meta-learning method, AdaMETA, for analyzing vibration signals obtained from bearings under different operating conditions. Compared to existing methods, AdaMETA provides three innovative contributions:

(a) Efficient Multi-scale Attention Feature Extraction Encoder (C-EMA): A feature extraction encoder based on efficient multi-scale attention (EMA) is introduced, capable of more efficiently capturing key features in fault signals and enhancing feature learning under limited sample conditions. By integrating multi-scale information, C-EMA adaptively adjusts attention to different scale features, thereby improving the model’s recognition accuracy across diverse fault patterns.
(b) Improved MAML Algorithm with Dynamically Adjusted Inner-Loop Learning Rate: To address the limitations of the traditional MAML algorithm, an improved mechanism for adjusting the inner-loop learning rate is proposed. By dynamically modifying the learning rate based on task complexity, the model can flexibly meet the learning requirements of different tasks, thereby enhancing the generalization performance for cross-domain tasks. This innovation not only optimizes the learning strategy but also increases the model’s adaptability when facing diverse task types.
(c) Validation of Cross-domain Generalization Capability from Multiple Source Domains to a Target Domain: To better align with real-world industrial applications, the dataset is divided into four domains, with three serving as source domains and one as the target domain. An experimental scheme is designed to test cross-domain generalization from multiple source domains to the target domain. This experimental setup verifies the model’s training effectiveness under multi-source domains and assesses its cross-domain generalization ability to the target domain. The model’s robustness and effectiveness are further evaluated through a sample-limited cross-domain diagnostic scenario and noise interference experiments.

The remainder of this paper is structured as follows: Section 2 introduces the fundamental theory of Model-Agnostic Meta-Learning (MAML) and the Efficient Multi-scale Attention Mechanism (EMA). Section 3 presents a detailed description of the proposed method and diagnostic procedures. The reliability of the proposed method is validated through multiple experimental sets in Section 4. Finally, Section 5 concludes the paper.

## 2. Theoretical Background

### 2.1. Meta-Learning

Meta-learning, often referred to as “learning how to learn,” is a training framework in which a model learns from a broad range of tasks, enabling it to quickly adjust to new tasks, even when only limited examples are available. The fundamental idea behind it is the acquisition of meta-knowledge—generalized strategies or patterns that work effectively across various tasks and can be easily adapted to new task requirements. In the case of small-sample learning, meta-learning methods provide notable advantages over traditional deep learning models, especially in contexts with sparse data. Conventional deep learning approaches for fault diagnosis typically rely on large amounts of labeled data and assume that the data distribution is consistent across the training (source) and testing (target) domains. However, collecting large-scale fault data and addressing domain shifts (e.g., variations in operational environments, loads, or machine types) are challenging in real-world industrial settings, leading to discrepancies between the source and target domains. Small-sample cross-domain fault diagnosis attempts to resolve this issue by utilizing a limited number of samples from the target domain while leveraging knowledge learned from the source domain. Meta-learning, as a potential solution, helps in acquiring generic representations across different tasks, thereby improving the model’s ability to generalize effectively to new domains with limited data.

### 2.2. Model-Agnostic Meta-Learning

A prominent optimization-based meta-learning approach is known as Model-Agnostic Meta-Learning (MAML), originally proposed by Finn et al. in 2017 [30]. The term “model-agnostic” indicates that MAML does not rely on any specific neural network architecture. Instead, it learns an optimal set of initial parameters, which can then be quickly adapted to a variety of new tasks using gradient descent methods. The primary objective of MAML is to identify the initial parameters θ such that the model requires only a few training steps to achieve effective performance on new tasks. In essence, MAML explicitly trains the initial weights to be quickly adaptable to new tasks. The optimization process involves a two-stage iterative procedure, often referred to as the inner and outer loops:

Inner loop (task adaptation): For each task $T_{i}$ drawn from the overall task distribution $P\left(T\right)$, the model begins with the current parameter set θ and performs one or more gradient updates using training data specific to that task. For instance, after one gradient update, the parameters adapted to task $T_{i}\;$ become:

(1) $$\theta_{i}^{\prime }=\theta -\alpha \nabla_{\theta }L_{T_{i}}^{train}\left(f_{\theta }\right)$$

where $L_{T_{i}}^{train}$ denotes the loss on the training dataset computed for task $T_{i}$, and α is the learning rate of the inner loop. This allows the task-specific parameter $\theta_{i}^{\prime }$ to be fine-tuned to better fit the training data for task $T_{i}$.

Outer loop (meta-optimization): After adaptation in the inner loop, MAML evaluates the adapted model parameters $\theta_{i}^{\prime }$ for each task separately and calculates a “meta-loss” using a meta-validation set for each task independently. This meta-loss measures how well the model adapts to each specific task. Next, MAML minimizes the sum of the meta-losses across all tasks by optimizing the initial parameter set θ. Formally, the meta-optimization objective is:

(2) $$\underset{\theta }{min}\;\sum_{T_{i}∼p\left(T\right)}\;L_{T_{i}}^{val}\left(f_{\theta_{i}^{\prime }}\right)=\underset{\theta }{min}\;\sum_{T_{i}}\;L_{T_{i}}^{val}\left(f_{\theta -\alpha \nabla_{\theta }L_{T_{i}}^{train}\left(f_{\theta }\right)}\right)$$

The above equation represents the total loss after one gradient adaptation step on each task. The gradient calculation used for optimization considers the variations introduced during the inner-loop adaptation and thus involves the computation of a second-order gradient. The initial parameters θ of the model are then updated as follows:

(3) $$\theta \leftarrow \theta -\beta \nabla_{\theta }\sum L_{T_{i}}^{val}\left(f_{\theta_{i}^{\prime }}\right)$$

**Figure 1:**
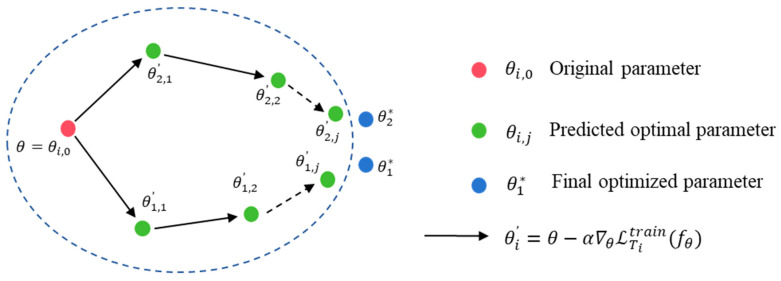
MAML parameter optimization process.

Here, β  denotes the meta-learning rate. Intuitively, this updating strategy adjusts the parameter θ to a region in the parameter space where small changes (e.g., a single gradient update) can lead to significant performance improvements on new tasks. The MAML parameter optimization process is shown in Figure 1.

### 2.3. Efficient Multi-Scale Attention (EMA)

Attention mechanisms in convolutional neural networks help dynamically highlight important features while minimizing the influence of irrelevant ones, significantly improving performance in tasks such as image recognition and reconstruction [39]. Typical attention mechanisms include channel-oriented attention (e.g., the widely used Compression and Excitation Module) and spatial-oriented attention [39], an example of which is the Convolutional Block Attention Module (CBAM) [40], which sequentially applies both types of attention. However, many existing channel attention techniques rely heavily on dimensionality reduction methods, often involving global pooling or bottleneck layers, which may inadvertently discard critical information. To address these issues, Ouyang et al. introduced the Efficient Multi-scale Attention (EMA) module [41], designed to retain information from each channel and efficiently model cross-channel interactions. Although initially proposed for lightweight image super-resolution and target detection, the core principles of EMA are sufficiently general to be applied to any task requiring multi-scale feature representation.

**Figure 2:**
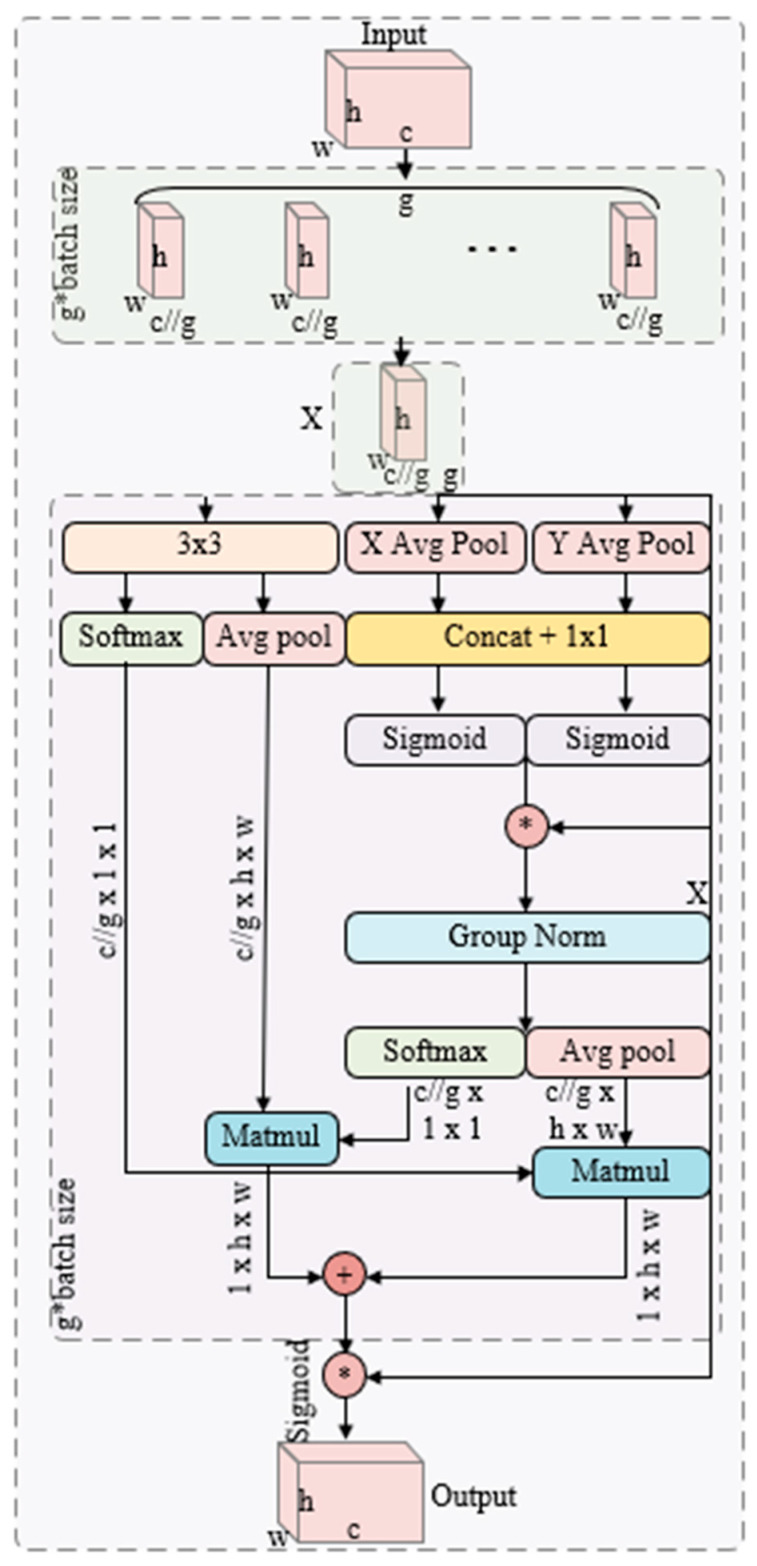
The structure of the EMA module, the * symbol represents Sigmoid activation function, and the + symbol indicates element-wise addition.

As shown in Figure 2, the specific EMA module processes feature maps simultaneously via parallel paths while maintaining the integrity of the channel information. Given an input feature tensor $U\in R^{C\times H\times W}$, C is used to denote the number of channels, and the EMA initially splits these channels into groups, effectively creating several sub-features, each of which captures a unique fragment of the channel domain. In practice, the channels are either partially reshaped into batch dimensions or split, i.e., splitting U into groups of G, denoted by ${U^{\left(g\right)}}_{g=1}^{G}$. Each group contains $C_{g}$ channels satisfying ∑gCg=C. This grouping approach distributes the spatial semantic information more evenly, capturing feature representations at different scales or receptive fields in the channel dimension. These grouped channels are then processed through parallel attention paths: in each path, global features are extracted to compute channel-specific attention weights, adjusting the strength of each group of features accordingly. Crucially, the EMA avoids significant dimensionality reduction during weight computation, thus preserving the detailed information in each channel. Mathematically, the channel attentional weights for each group g can be expressed as $w^{\left(g\right)}\in R^{C_{g}}$. The attention vectors are computed by globally pooling the sub-features $U^{\left(g\right)}$ and subjecting them to a learnable transformation (e.g., a fully connected layer or a $\left(1\times 1\right)$ convolution). Each weighted sub-feature is then represented as $U^{\prime \left(g\right)}=w^{\left(g\right)}⊙U^{\left(g\right)}$, where ⊙ denotes channel-wise multiplication.

## 3. The Proposed Method

### 3.1. Description of Cross-Domain Scenarios

Cross-Domain Learning (CDL) aims to improve the generalization ability of models on the Target Domain by migrating knowledge between different but related data domains. Its core challenge lies in the inter-domain distribution difference, the data distributions of the source domain and target domain $Psource\left(X,Y\right)\neq Ptarget\left(X,Y\right)$, which leads to a significant degradation in the performance of direct migration models.

Formal Definition:

- Domain: consists of the joint distribution $P\left(X,Y\right)$ of the data space X and label space Y.
- Task: the mapping from Input X to Output Y,f:X→Y.
- Cross-domain scenario: given a source domain $D_{s}=\{(x_{i}^{s},y_{i}^{s}){\}}_{i=1}^{N_{s}}\;$ and a target domain $D_{t}=\{(x_{j}^{t},y_{j}^{t}){\}}_{j=1}^{N_{t}}$ satisfies $Ps\left(X,Y\right)\neq Pt\left(X,Y\right)$, and usually Nt≪Ns.

Small-sample cross-domain fault diagnosis of bearings can address scenarios with few fault samples under different operating conditions, but the inter-domain differences may contain both covariate bias and conceptual bias, making distributional alignment difficult, and the small-sample target domains are prone to leading to overfitting of the model with noisy or domain-specific features.

### 3.2. Construction of the C-EMA Feature Encoder

**Figure 3:**
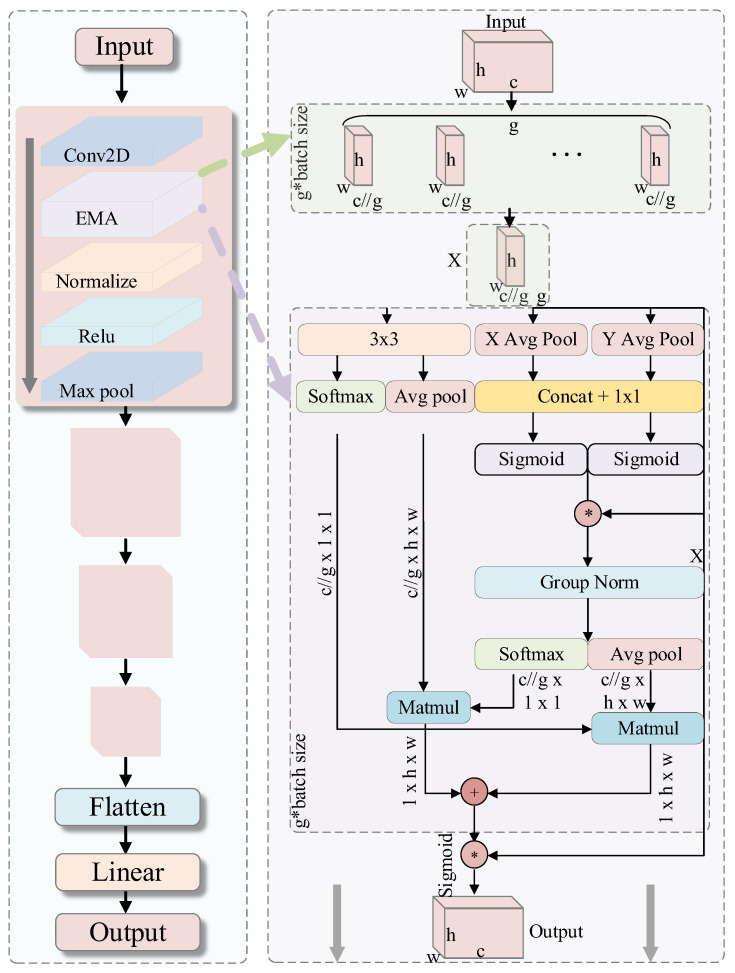
The structure of the C-EMA module. The * symbol represents Sigmoid activation function, and the + symbol indicates element-wise addition.

For small-sample cross-domain scenarios, this study proposes a hierarchical feature encoder that integrates a novel Efficient Multi-scale Attention (EMA) module, designed to enhance cross-scale feature integration while maintaining computational efficiency. The network’s overall structure retains the hierarchical stacking of classical CNNs, with the EMA module strategically placed between the convolutional and normalization layers. This positioning allows the EMA module to directly modulate the original feature response. The design provides the network with three key properties: capturing multi-scale cross-channel dependencies, preserving spatial information through shallow integration, and maintaining gradient flow via residual connectivity. Unlike traditional convolutional feature processing, EMA adaptively enhances features in both the channel and spatial dimensions through local and global pooling, attentional weighting, and group normalization. As illustrated in Figure 3, the network architecture consists of four core components:

(a) A multilayer convolutional backbone;
(b) The EMA attention mechanism;
(c) A batch normalization layer;
(d) A nonlinear activation layer.

In each convolutional block (ConvBlock), a 3 × 3 convolution is performed to extract base features, followed by adaptive rescaling of the convolutional outputs through multi-scale attention via the EMA module. Next, Batch Normalization (BN) with the ReLU activation function is applied to stabilize the feature distribution for the downstream layers. The optional MaxPool operation is then used to downsample, expand the receptive field, and reduce the feature map size. At the top layer, either global average pooling or a linear classifier can be selected to generate the final output, depending on the specific task requirements. The structure and details of each layer are summarized in Table 1.

Through the joint attention mechanism of global and local pooling, EMA effectively integrates dependencies between distant and neighboring pixels, enhancing the network’s capacity to identify complex failure modes and subtle variations, which is of paramount importance. Group normalization and attentional fusion help suppress noisy activations in the feature space, reducing distributional fluctuations caused by random batches in small-sample scenarios. In this work, the channel number C of the feature maps input to the EMA module is 64. We set the group number G to 4, resulting in each subgroup containing C_g = 16 channels. This configuration strikes a balance between capturing diverse multi-scale features and maintaining computational efficiency, which is a common and effective practice for feature maps of this scale [41].

### 3.3. Dynamic Task-Aware Inner-Loop Learning Rate α

In small-sample cross-domain fault diagnosis, It is evident that there are significant disparities in the data distributions between the source and target domains, which consequently result in a decline in the performance of the model during the process of migration. The original MAML struggles to adapt to the heterogeneity of different domain tasks. In this paper, we propose dynamic tuning of the inner-loop learning rate (DT-MAML), based on the core idea of gradient-sensitive dynamic adjustment. The learning rate can be adaptively adjusted according to the task gradient paradigm:

(4) $$\alpha_{\tau }=\frac{\alpha }{∥\nabla L_{\tau }\left(\theta \right){∥}_{2}+\epsilon }$$

where

$∥\nabla L_{\tau }\left(\theta \right){∥}_{2}$ is the $L_{2}$ norm of the gradient vector, and ϵ is a very small constant (e.g., 10^−8^) to prevent numerical instability. The update rule is amended to:
  (5) $$\theta^{\prime }=\theta -\alpha_{\tau }\nabla_{\theta }L_{T_{i}}^{train}\left(f_{\theta }\right)$$

The learning rate is decreased when encountering high-gradient tasks (e.g., large cross-domain differences or complex failure modes) to prevent oscillations caused by overshooting parameter updates. Conversely, the learning rate is increased when encountering low-gradient tasks (e.g., similar domains or simple failure modes) to accelerate convergence. This method has been demonstrated to have a significant impact on the robustness of the model with regard to unknown domain tasks.

The dynamic inner-loop learning rate mechanism naturally constrains the magnitude of parameter updates:

(6) $$∥\Delta \theta ∥=\alpha_{\tau }∥\nabla L_{\tau }\left(\theta \right){∥}_{2}\leq \frac{\alpha }{1+\epsilon /∥\nabla L_{\tau }\left(\theta \right){∥}_{2}}$$

**Figure 4:**
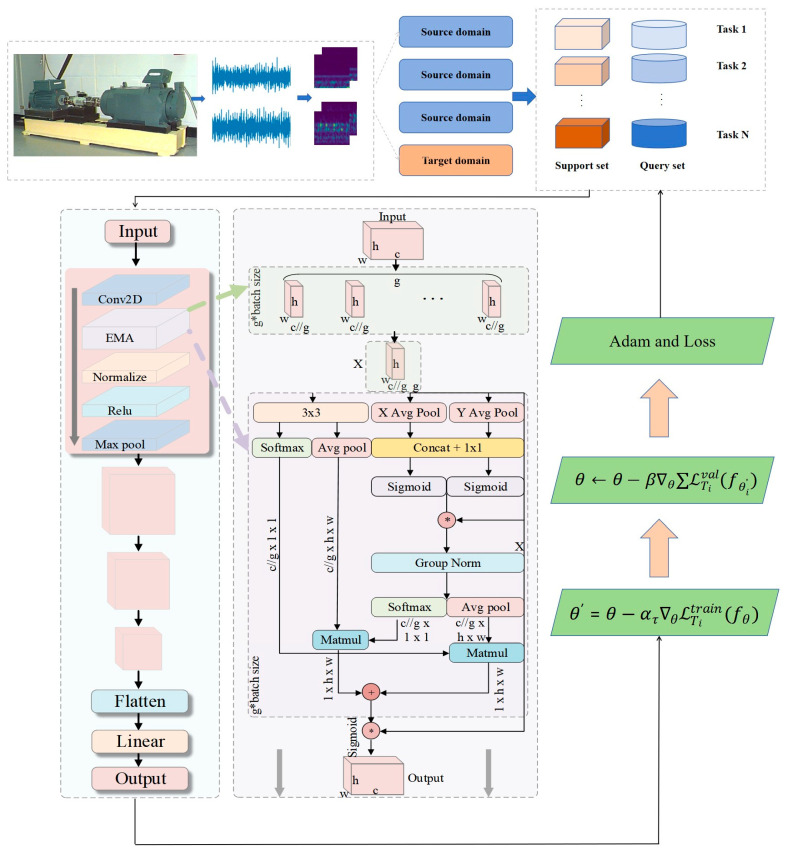
The overall procedure of the AdaMETA diagnostic model. The * symbol represents Sigmoid activation function, and the + symbol indicates element-wise addition.

Even if there are abnormal gradients (e.g., noisy samples), the update amount is still limited to a reasonable range, which improves the stability of small-sample training and has almost zero additional overhead, significantly improving the cross-domain small-sample performance. The base inner-loop learning rate α is set to 0.01, which is a standard value in MAML algorithms for rapid task adaptation [31]. This base value is then dynamically normalized by the gradient norm as formulated in Equation (4). The pseudo-code of the training process of the DT-MAML algorithm is shown in Algorithm 1, and the overall architecture of the proposed method is illustrated in Figure 4.
**Algorithm 1**: The training algorithm of the DT-MAML network**Require:** Dataset *D*, number of classes *N*, shots *K*, initial meta-parameters *θ*, inner-loop learning rate *α* = 0.01, outer-loop Adam optimizer, iterations *T***Ensure:** Optimized meta-parameters θ*  1:Initialize *θ* randomly  2:**for** iteration t = 1 **to** T **do**  3:$\;\;\mathrm{Sample}\;\mathrm{task}\;\mathrm{batch}\;\{\tau_{i}{\}}_{i=1}^{n}$   ΔEach task is N-way K-shot  4:    $\mathrm{Initialize}\;\nabla_{\theta }L_{meta}\leftarrow 0$  5:    **for** each task τi **do**  6:      Sample N classes from D  7:     For each class, sample K $\mathrm{support}\;D_{\tau i}^{spt}$ and Q $\mathrm{query}\;D_{\tau i}^{qry}$  8:        $\mathrm{Clone}\;\theta_{i}^{\prime }\leftarrow \theta$       ΔInner-loop adaptation  9:    **for** inner step k = 1 **to** Kinner **do**10:        Compute cross-entropy loss on support set:$$L_{\tau_{i}}^{\mathrm{spt}}=-\frac{1}{NK}\sum_{\left(x,y\right)\in D_{\tau_{i}}^{\mathrm{spt}}}\sum_{c=1}^{N}y_{c}\log f_{\theta_{i}^{\prime }}{\left(x\right)}_{c}$$11:        $\mathrm{Compute}\;\mathrm{gradients}:\;g_{i}\leftarrow \nabla_{\theta_{i}^{\prime }}L_{\tau_{i}}^{\mathrm{spt}}$12:        $\mathrm{Compute}\;\mathrm{gradient}\;\mathrm{norm}:\;||g_{i}{\left|\right|}_{2}\leftarrow \sqrt{\sum_{j}{\left(g_{i}^{\left(j\right)}\right)}^{2}}$13:       $\mathrm{Adjust}\;\mathrm{learning}\;\mathrm{rate}:\;\alpha_{\tau_{i}}\leftarrow \alpha /||g_{i}{\left|\right|}_{2}+\epsilon$14:        $\mathrm{Update}\;\mathrm{parameters}:\;\theta_{i}^{\prime }\leftarrow \theta_{i}^{\prime }\;-\;\alpha_{\tau_{i}}g_{i}$15:     **end for**16:    Compute query loss:$$L_{\tau_{i}}^{qry}=-\frac{1}{N\;Q}\sum_{\left(x,y\right)\in D_{\tau_{i}}^{qry}}\sum_{c=1}^{N}y_{c}\;\log f_{\theta_{i}^{\prime }}{\left(x\right)}_{c}$$17:    $\mathrm{Compute}\;\mathrm{meta}\text{-}\mathrm{gradient}:\;\nabla_{\theta }L_{\mathrm{qry}}^{\tau_{i}}\leftarrow \nabla_{\theta }L_{\mathrm{qry}}^{\tau_{i}}$18:    $\mathrm{Accumulate}\;\mathrm{gradients}:\;\nabla_{\theta }L_{\mathrm{meta}}\leftarrow \;\nabla_{\theta }L_{\mathrm{meta}}\;+\;\frac{1}{n}\;\nabla_{\theta }L_{\mathrm{qry}}^{\tau_{i}}$19:  **end for**20:   $\mathrm{Update}\;\mathrm{meta}\text{-}\mathrm{parameters}:\;\theta \;\leftarrow \;\mathrm{Adam}\left(\theta ,\;\nabla_{\theta }L_{\mathrm{meta}}\right)$21:**end for**22:**Return optimized meta-parameters** θ* ← θ

## 4. Experimental Results and Analysis

### 4.1. Dataset Processing

#### 4.1.1. Overview of the CWRU Dataset

**Figure 5:**
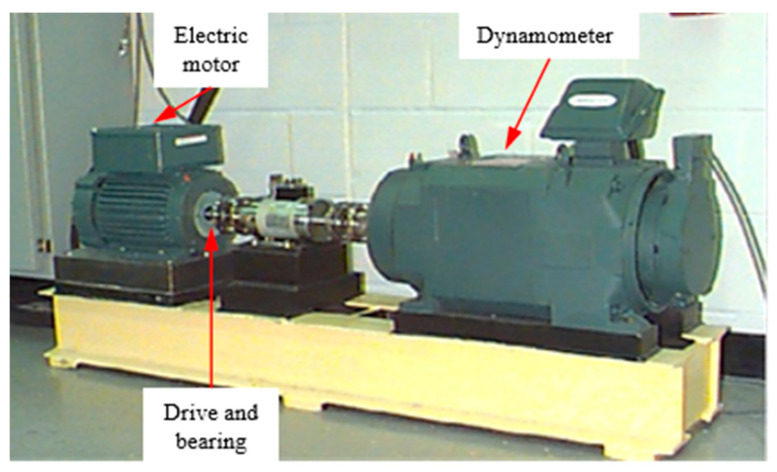
The bearing test device.

The CWRU (Case Western Reserve University Bearing Data Center) bearing failure dataset is one of the most commonly used public datasets in the field of health monitoring of rotating machinery [42] and is widely used for the validation of vibration signal-driven fault diagnosis algorithms. The data are collected by piezoelectric accelerometers mounted at the drive end (DE) and fan end (FE) with sampling frequencies of 12 kHz and 48 kHz. The experimental platform, as shown in Figure 5, uses 2 hp three-phase induction motors, with speeds corresponding to four load conditions (0 hp, 1 hp, 2 hp, and 3 hp) and rated speeds of approximately 1797 rpm, 1772 rpm, 1750 rpm, and 1730 rpm, respectively. The failure types cover Normal and three typical defects—Inner Ring (IR), Outer Ring (OR), and Rolling Element (RE), each with three damage sizes: 0.007″, 0.014″, and 0.021″, for a total of nine failure states (see Table 2). The defects are accurately hole-made by electric discharge machining (EDM), which ensures the consistency of fault depth and location, thus ensuring that the dataset has a high degree of confidence in terms of controllability of working conditions and experimental reproducibility. The raw data are stored in the form of time-domain vibration signals without any preliminary processing and are suitable for the extraction of features and modeling methods in the time domain, frequency domain, and time–frequency domain.

#### 4.1.2. Experimental Data Partitioning and Small-Sample Cross-Domain Settings

To capture load-dependent distribution shifts, the CWRU data are split into four load domains (0, 1, 2, 3 hp), each with 10 classes (1 normal, 9 faults). In cross-domain few-shot detection, one domain is randomly chosen as the target $D_{t}$; the remaining three are merged as the source $D_{s}$. We train fully supervised on Ds. In $D_{t}$, only K samples per class (K ≤ 5) are used for adaptation, and the rest are used for testing, simulating scarce target-condition data. This design preserves load-induced statistical differences and avoids fault-type confounds, providing a clearer test of generalization and robustness.

**Figure 6:**
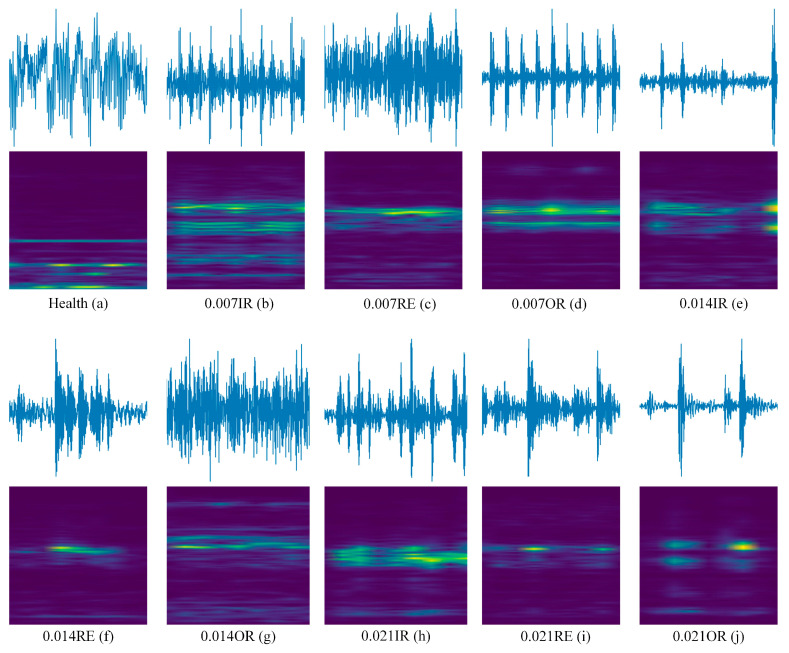
Under 1772 ram: (**a**) healthy; (**b**–**j**) correspond to the original vibration signals and time–frequency diagrams of IR, RE, and OR at damage levels of 0.007/0.014/0.021 mm, respectively.

Raw vibration signals are segmented with a 1024-sample sliding window (≈1024/fs, covering ≥ 2 rotor cycles) and 50% overlap to augment data and limit inter-sample correlation while preserving frequency resolution. After segmentation, short-time Fourier transforms (STFTs) produce fixed-size time–frequency images that capture local transients and global spectral patterns (see Figure 6). Compared with pure time–domain features, these representations are more sensitive to cross-load distribution shifts and offer a stronger basis for few-shot cross-domain diagnosis.

### 4.2. Comparison of Algorithms in Different Cross-Domain Scenarios

**Figure 7:**
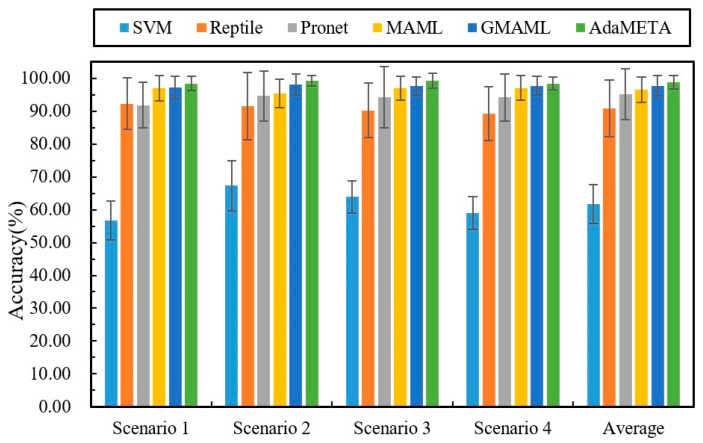
Results of different methods in four cross-domain scenarios.

This section evaluates the performance of the AdaMETA diagnostic model across four distinct low-shot cross-domain scenarios, with the scenario details provided in Table 3. For each sub-task, which follows the ‘10-way 5-shot’ configuration, five samples from each class in the source domain are randomly selected to construct the task. The diagnostic model, once trained, is tested under different load conditions in the target domain. The results for all methods are shown in Table 4 and Figure 7.

The results show that, in comparative experiments across four typical load scenarios (Scenario 1 to Scenario 4), the proposed method achieves the highest classification accuracy across all test conditions with the smallest variation. Specifically, the accuracy rates for Scenarios 1 to 4 were 98.37 ± 2.17%, 99.16 ± 1.62%, 99.26 ± 2.31%, and 98.39 ± 1.88%, respectively, with an average accuracy of 98.8 ± 1.99%. The following results were obtained through comparison.

The comparative results demonstrate that, in the few-shot cross-domain fault diagnosis knowledge transfer setting, meta-learning frameworks significantly outperform traditional machine learning and conventional deep learning methods. Using Support Vector Machine (SVM) as a benchmark, the average diagnostic accuracy across the four load scenarios is only 61.75%, with the best performance in Scenario 2 at 67.35%. In contrast, under the same conditions, the four meta-learning models—Reptile, ProNet, MAML, and GMAML—achieved average accuracies of 90.8%, 95.2%, 96.56%, and 97.68%, respectively. This performance gap arises because meta-learning algorithms emphasize cross-task transfer and rapid adaptation during training, rather than overfitting to individual samples, enabling efficient and robust fault identification even in cases where target-domain samples are scarce.

**Figure 8:**
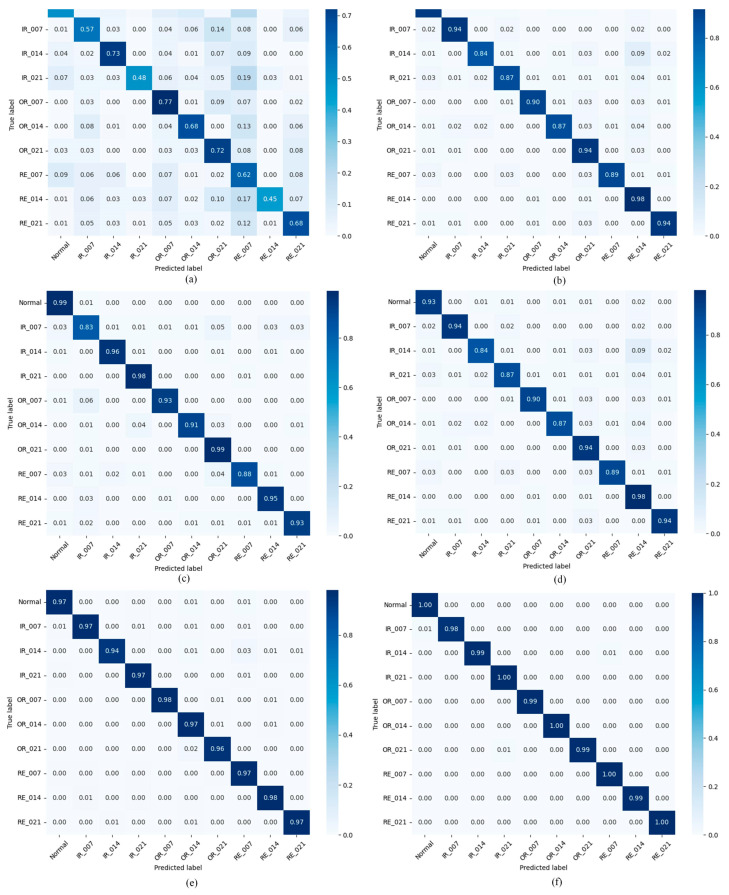
Confusion matrix of comparison methods in Scenario 2: (**a**) SVM; (**b**) Reptile; (**c**) ProNet; (**d**) MAML; (**e**) GMAML; (**f**) proposed method.

Compared with typical meta-learning baselines such as Reptile, ProNet, and MAML, the proposed method achieves higher fault identification accuracy across four cross-load transfer scenarios. In these scenarios, the average accuracy improved by 8.0% over Reptile, 3.60% over ProNet, and 2.24% over MAML. Compared to the latest GMAML method, the proposed method improved by 1.12%. These gains stem from the method’s ability to efficiently extract shared diagnostic priors across multiple source-domain tasks and leverage a rapid adaptation mechanism to fully exploit the information potential of sparse target-domain samples, significantly enhancing cross-domain generalization. As illustrated in Figure 8, the 3D confusion matrix presents the specific classification results of the various methods in Scenario 2, demonstrating that the proposed method accurately identifies samples across categories.

### 4.3. Ablation Experiments of the Proposed Method

**Figure 9:**
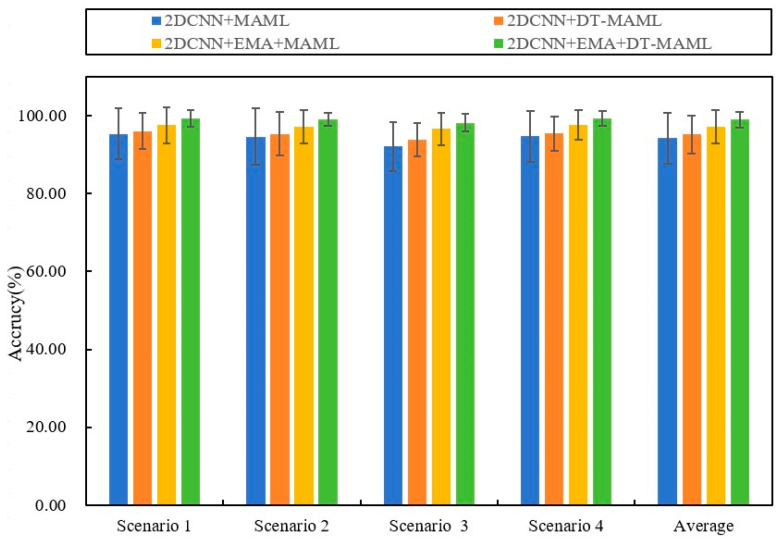
The results of the ablation experiments for various methods across four different scenarios.

To rigorously assess the contribution of each component to overall performance, this study designed ablation experiments evaluating four model combinations across four scenarios; the results are shown in Figure 9, and the specific accuracy is shown in Table 5.

The experimental data revealed significant performance differences across scenarios. In Scenario 1, 2DCNN+EMA+DT-MAML performed best, with an accuracy of 98.37 ± 2.17%, significantly outperforming the other methods. A similar trend was observed in Scenario 2, where 2DCNN+EMA+DT-MAML attained 99.16 ± 1.62% accuracy, again exceeding the other combinations. In Scenarios 3 and 4, 2DCNN+EMA+DT-MAML continued to perform strongly, achieving 99.26 ± 2.31% and 98.39 ± 1.88% accuracy, respectively, and remaining significantly higher than the models that did not include EMA or DT.

Further analysis of average performance showed that the 2DCNN-based model combining EMA and DT (i.e., 2DCNN+EMA+DT-MAML) achieved an average accuracy of 99.04 ± 1.99% across the four scenarios, significantly outperforming the other combinations. This result confirms the importance and effectiveness of EMA and DT in improving the model’s generalization and robustness. Additionally, compared with using DT or EMA alone, their joint use produced a synergistic effect, further improving model performance.

In summary, these ablation experiments clearly demonstrate the key role of EMA and DT-MAML in enhancing the performance of the proposed method and validate the effectiveness of their joint application.

### 4.4. Effect of Dynamic Inner-Loop Learning Rate α on Diagnostic Performance

**Figure 10:**
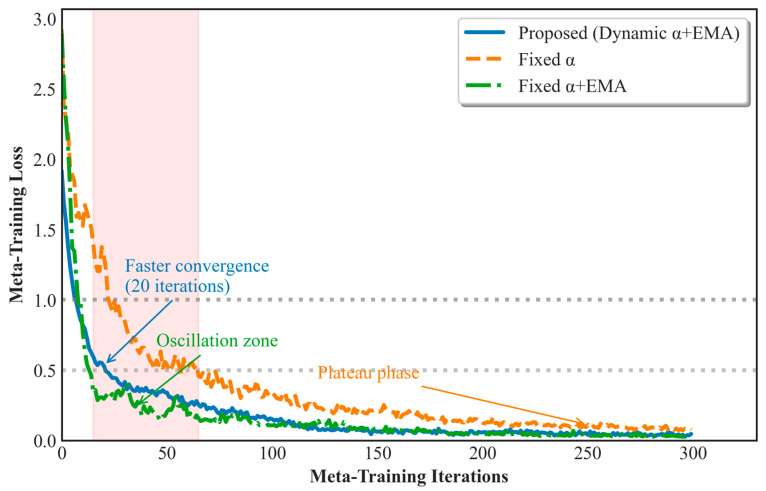
Loss curves for the three methods after 300 iterations.

To evaluate the impact of the dynamic inner-loop learning rate α on the model’s convergence performance, this study conducted three comparative experiments: fixed α, fixed α combined with the EMA module (fixed α + EMA), and fixed α + EMA combined with dynamic α (fixed α + EMA + dynamic α, i.e., the method proposed in this paper). The detailed information is shown in Table 6. The experimental results, as shown in Figure 10, demonstrate that the dynamic α method (blue line) rapidly reduces the loss value to 1.0 after only 15 iterations, approximately 40% faster than the fixed α+EMA method (green line), highlighting the significant role of dynamic α in accelerating convergence during the early stages of training.

Notably, the fixed α + EMA method exhibits noticeable loss fluctuations and oscillations between 15 and 65 iterations (red region), reflecting instability in the optimization process due to task conflicts, which limits the model’s rapid convergence early on. In contrast, the model using a fixed α (orange line) did not exhibit oscillations but reached a plateau after 200 iterations, with the loss value stabilizing around 0.3 and failing to decrease further. This suggests that a fixed learning rate lacks an effective adjustment mechanism, preventing optimization in the later stages of training.

In contrast, the dynamic α scheduling strategy effectively mitigates these issues, avoiding oscillations while continuously driving model optimization in the later stages of training, ultimately reducing the loss value to a minimum of 0.1. These results demonstrate the critical role of dynamic learning rate α scheduling in improving the model convergence speed, avoiding oscillations, and driving optimization in the later stages of training, which positively impacts the model’s generalization performance. The experimental results confirm the significant value of dynamic learning rate α in cross-domain fault diagnosis tasks from both theoretical and practical perspectives.

### 4.5. Comparison of Algorithm Effect in Different Noise Environments

In order to verify the robustness and generalization ability of the proposed small-sample cross-domain bearing fault diagnosis method in real industrial environments, this study further carried out experimental analyses under noise interference conditions. Specifically, Gaussian white noise of different intensities was artificially added to the bearing vibration signals to simulate the sensor measurement errors and environmental disturbances in the real production environment. Gaussian white noise is a random signal with uniform spectrum and obeys Gaussian distribution, and its probability density function is defined as: $f\left(x\right)=\frac{1}{\sqrt{2\pi }\sigma }e^{-\frac{(x-\mu {)}^{2}}{2\sigma^{2}}}$, where μ denotes the mean of the noise and $\sigma^{2}$ denotes the variance.

**Figure 11:**
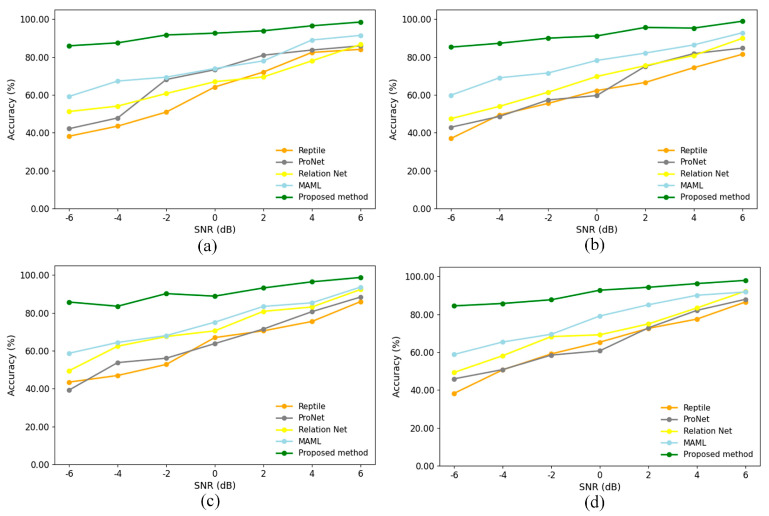
Accuracy rate after 1000 iterations of different methods with different signal-to-noise ratios in four scenarios: (**a**–**d**) Scenarios 1–4.

In the experiments, the diagnostic accuracy of the proposed method is compared and analyzed with four classical methods, namely, Reptile, ProNet, RelationNet, and MAML, in multiple cross-domain scenarios using different signal-to-noise ratios (SNRs) from −6 dB to 6 dB. The experimental results are shown in Figure 11. As the noise intensity increases (i.e., the SNR value decreases), the accuracy of each diagnostic method tends to decrease. However, the method proposed in this paper consistently exhibits stronger noise immunity and is able to maintain the highest diagnostic accuracy under all noise levels with a slower and more stable decreasing trend. This indicates that the method proposed in this paper can effectively suppress noise interference, demonstrates significant robustness and generalization performance, and is more suitable for the complex and changing environments in real industrial scenarios.

### 4.6. T-SNE Visualization

**Figure 12:**
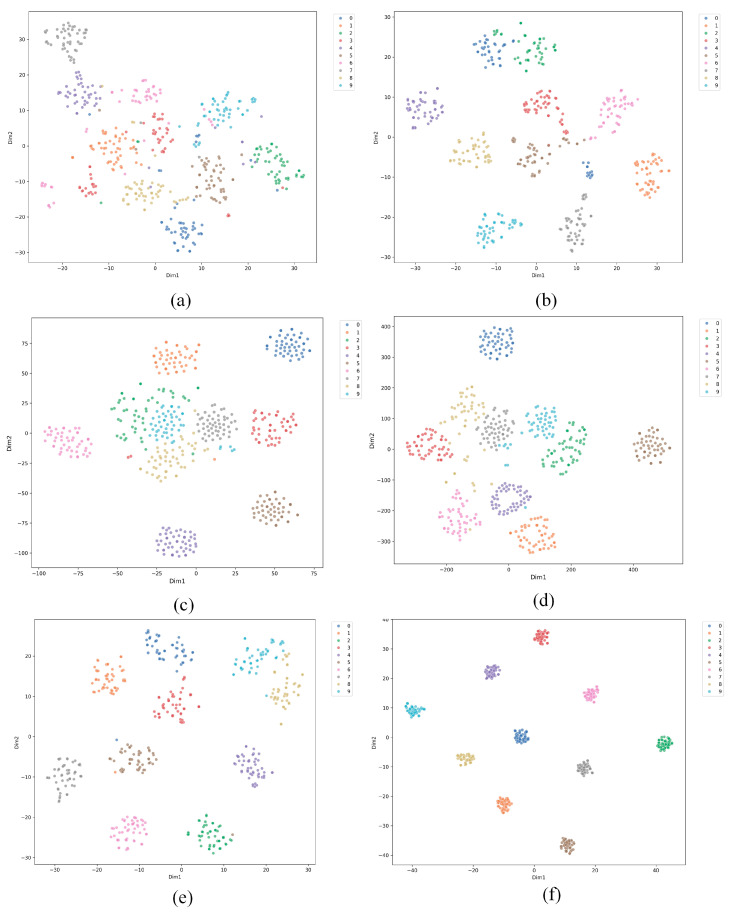
Visualization of feature maps for six methods in Scenario 2: (**a**) SVM; (**b**) Reptile; (**c**) ProNet; (**d**) MAML; (**e**) GMAML; (**f**) proposed method.

Figure 12 presents the t-SNE results for six methods applied to small-sample cross-domain fault diagnosis of bearings. Each sub-figure shows a different method with data points colored by class. (a) shows the results for SVM, with widely spread data points and some class overlap, indicating difficulty in handling the task. (b) shows the results for Reptile, with more distinct clusters but still some class mixing, suggesting better performance than SVM but challenges with small samples. (c) and (d) show the results for Prototypical Networks (ProtoNet) and Relation Networks (RelationNet), both showing more compact clusters and reduced overlap, though some intersections remain. (e) shows the results for MAML, with better separation and less overlap, indicating strong adaptability to small-sample cross-domain tasks. (f) shows the results for the proposed method, which achieves the best clustering with minimal overlap, demonstrating superior performance in handling small-sample cross-domain fault diagnosis. Overall, while traditional methods such as SVM struggle with class separation, newer meta-learning approaches, especially the proposed method, significantly improve the handling of small-sample cross-domain tasks.

### 4.7. Comparison of Attention Mechanisms

To validate the superiority of the Efficient Multi-scale Attention (EMA) module in few-shot cross-domain diagnostic tasks and address the reviewers’ suggestions, this section presents comparison experiments with mainstream attention mechanisms. Under the identical AdaMETA framework, network architecture, and training settings, we replace the C-EMA module with two widely used and classical attention mechanisms for comparison:

Squeeze-and-Excitation Network (SENet) [43]: A classic channel attention mechanism that performs squeezing via global average pooling and constructs inter-channel dependencies using fully connected layers.

Convolutional Block Attention Module (CBAM) [40]: A hybrid attention mechanism that sequentially applies channel attention followed by spatial attention.

The experiments are conducted in the most representative cross-load Scenario 2 (source domains: D_0_, D_1_, D_3_ → target domain: D_2_), with the task setting of 10-way 5-shot. All comparison methods employ the same dynamic learning rate strategy (DT-MAML) to ensure fairness. The comparison results are shown in Table 7.

The analysis of Table 7 leads to a clear conclusion: the EMA module we adopted achieves the best diagnostic performance while introducing the lowest computational overhead.

Performance Advantage: The accuracy and stability of EMA are significantly higher than those of SENet and CBAM. We attribute its advantage primarily to the fact that EMA does not involve dimensionality reduction and employs multi-scale grouping. SENet uses fully connected layers for dimensionality reduction in channel attention, which may lead to information loss. In contrast, the EMA module avoids any form of dimensionality reduction, preserving the integrity of channel information to the greatest extent. Additionally, by processing features through grouping and integrating multi-scale receptive fields, EMA is more flexible in capturing multi-scale patterns in fault signals than the single-scale CBAM.

Efficiency Advantage: As shown in Table 7, the additional parameter count (ΔParams) and computational load (ΔFLOPs) of the EMA module are much lower than those of CBAM and significantly lower than SENet. This advantage stems from EMA’s compact group structure and parallel path design, which achieves powerful attention effects through efficient intra-group cross-channel interaction, without requiring complex submodules (such as the spatial attention in CBAM) or fully connected layers (such as SENet).

In conclusion, this comparison experiment robustly demonstrates, from both performance and efficiency perspectives, that the EMA module is a more competitive choice than SENet and CBAM for the few-shot cross-domain bearing fault diagnosis task in this paper, achieving the best balance between performance and complexity.

### 4.8. Cross-Sensor Location Generalization Capability Verification Experiment

To further validate the generalization ability of the AdaMETA framework under different distribution shifts, this section presents a novel and more challenging experiment: fault diagnosis across sensor locations. This experiment simulates a common industrial scenario, where a model trained at one location (e.g., the drive end) is required to effectively diagnose faults at a different location (e.g., the fan end) with only a few samples.

#### 4.8.1. Experimental Setup and Data Partitioning

This experiment is based on the CWRU dataset, utilizing the vibration data collected simultaneously from both the drive end (DE) and fan end (FE). Although the same bearing system is monitored, inherent differences in vibration signals arise due to variations in the mechanical sensor mounting positions, including differences in signal propagation paths, attenuation characteristics, and signal-to-noise ratios. These variations result in significant data distribution shifts, providing an ideal and realistic “cross-domain” validation platform.

Source Domain ($D_{s}$): The data collected from the drive end (DE) under four different load conditions (0, 1, 2, 3 hp) are selected. This forms a diverse source domain designed to help the model learn fault features at the drive end that are independent of load conditions and can generalize across the drive end.

Target Domain ($D_{t}$): The data collected from the fan end (FE) under the same four load conditions are selected. The key setup here is that, in the target domain, we simulate an extreme small-sample scenario, where only five samples (i.e., 5-shot) are provided for each fault category (10 categories in total) to adapt the model. The remaining fan end samples are used for testing.

Task Construction: We follow the 10-way 5-shot meta-learning task format as outlined in Section 4.1.2. Each training task is randomly sampled from the diverse loads in the source domain (DE), while testing is conducted on the small sample set from the target domain (FE) for adaptation and evaluation. The data preprocessing pipeline (1024-sample-length sliding window and STFT converted to time-frequency spectrograms) is consistent with the main experiment to ensure fairness in comparisons.

This “DE -> FE” transfer setup is significantly more challenging than the previous cross-load transfer. It not only involves load variation but also introduces more fundamental signal characteristic changes due to the physical location difference of the sensors.

#### 4.8.2. Cross-Location Diagnostic Results and Analysis

We compared the proposed AdaMETA method with a series of baseline methods in this new scenario, and the results are shown in Table 8.

The analysis of Table 8 leads to the following conclusions:

(a) Task Challenge: The average accuracy of all methods shows a significant decline compared to the cross-load experiment in Section 4.2. This confirms that the distribution differences caused by cross-sensor locations are more severe than simple load variations.
(b) Outstanding Generalization of AdaMETA: The proposed AdaMETA method still achieves the best performance in this new scenario, with an accuracy of 97.63%, which is significantly higher than the other comparison methods. Compared to the strong baseline GMAML, our method provides an improvement of approximately 2.75%.
(c) Stability Demonstration: AdaMETA also achieves the lowest standard deviation (3.29%), indicating that our method exhibits stronger robustness and stability when facing complex distribution shifts caused by location changes, making it less sensitive to task random sampling.

## 5. Conclusions

In conclusion, this paper has presented AdaMETA, an adaptive meta-learning framework that effectively addresses the challenge of few-shot bearing fault diagnosis under varying operational conditions. By integrating an Efficient Multi-scale Attention (EMA) module for enhanced feature extraction and a novel dynamic task-aware mechanism (DT-MAML) for adaptive inner-loop optimization, the framework achieves robust cross-domain generalization.

Comprehensive experimental evaluations on the CWRU dataset demonstrate the superiority of AdaMETA, which attained a peak accuracy of 99.26% in cross-load scenarios and exhibited strong performance in the newly added cross-sensor location task. Ablation studies and noise robustness tests further confirmed that the synergistic design of the EMA module and the dynamic learning rate strategy are pivotal to the model’s high accuracy and stability.

Overall, AdaMETA provides a powerful and practical solution for fault diagnosis in data-scarce industrial environments. Future work will focus on validating the framework on more diverse industrial datasets and extending it to address more complex fault patterns, such as compound faults and evolving fault severities.

## Figures and Tables

**Table 1:** Structure and size dimensions of each layer of the C-EMA.

Layer Type	Input Size	Output Size	Operation
ConvBlock 1	(3, 32, 32)	(64, 16, 16)	Convolution→EMA→BatchNorm→ReLU→Max Pooling
ConvBlock 2	(64, 16, 16)	(64, 8, 8)	Convolution→EMA→BatchNorm→ReLU→Max Pooling
ConvBlock 3	(64, 8, 8)	(64, 4, 4)	Convolution→EMA→BatchNorm→ReLU→Max Pooling
ConvBlock 4	(64, 4, 4)	(64, 2, 2)	Convolution→EMA→BatchNorm→ReLU→Max Pooling
Flatten	(64, 2, 2)	(256)	Flatten to 1D vector
Linear Layer	(256)	(output_size)	Fully connected layer, output is output_size

**Table 2:** CWRU dataset domain classification and load details.

Domain Number	Load Gear (hp)	Motor Speed (rpm)	Includes Categories
D_1_	0 hp	≈1797 rpm	Normal + 9 Faults
D_2_	1 hp	≈1772 rpm	Normal + 9 Faults
D_3_	2 hp	≈1750 rpm	Normal + 9 Faults
D_4_	3 hp	≈1730 rpm	Normal + 9 Faults

**Table 3:** Description of four cross-domain scenarios.

Cross-Domain Scenario	Source-Domain	Target-Domain
Scenario 1	$D_{0}D_{1}D_{2}$	$D_{3}$
Scenario 2	$D_{0}D_{1}D_{3}$	$D_{2}$
Scenario 3	$D_{0}D_{2}D_{3}$	$D_{1}$
Scenario 4	$D_{1}D_{2}D_{3}$	$D_{0}$

**Table 4:** Diagnostic accuracy of different methods in four cross-domain scenarios (percentage).

Method	Scenario 1	Scenario 2	Scenario 3	Scenario 4	Average
SVM	56.72 ± 5.95	67.35 ± 7.61	63.88 ± 4.96	59.04 ± 5.05	61.75 ± 5.89
Reptile	92.23 ± 7.83	91.52 ± 10.32	90.21 ± 8.35	89.24 ± 8.27	90.8 ± 8.69
ProNet	91.82 ± 6.92	94.65 ± 7.64	94.18 ± 9.26	94.15 ± 7.15	95.2 ± 7.74
MAML	96.92 ± 3.84	95.33 ± 4.26	96.98 ± 3.72	97.02 ± 3.79	96.56 ± 3.9
GMAML	97.26 ± 3.42	98.12 ± 3.11	97.61 ± 2.74	97.73 ± 2.93	97.68 ± 3.05
Proposed method	98.37 ± 2.17	99.16 ± 1.62	99.26 ± 2.31	98.39 ± 1.88	98.8 ± 1.99

**Table 5:** Diagnostic accuracy of different methods in the “10-way 5-shot” scenario in Scenario 2 (percentage).

Method	Scenario 1	Scenario 2	Scenario 3	Scenario 4	Average
2DCNN+MAML	95.36 ± 6.56	94.72 ± 7.23	92.16 ± 6.29	94.81 ± 6.51	94.26 ± 6.65
2DCNN+DT-MAML	96.09 ± 4.67	95.38 ± 5.64	93.16 ± 4.36	95.48 ± 4.39	95.21 ± 4.77
2DCNN+EMA+MAML	97.58 ± 4.69	97.25 ± 4.32	96.70 ± 4.21	97.66 ± 3.84	97.29 ± 4.26
2DCNN+EMA+DT-MAML	98.37 ± 2.17	99.16 ± 1.62	99.26 ± 2.31	98.39 ± 1.88	98.9 ± 1.99

**Table 6:** Parameter settings for different control groups.

Comparison Group	Definition	Learning Rate Strategy	Attention Module	Dataset Partitioning
Baseline	Standard MAML	Fixed α (0.01)	None	CWRU Four Domains
Proposed	Our full model	Dynamic α	EMA	CWRU Four Domains
Ablation	Ablation of dynamic α	Fixed α (0.01)	EMA	CWRU Four Domains

**Table 7:** Performance comparison of different attention mechanisms in cross-domain diagnostic tasks (Scenario 2, 10-way 5-shot).

Attention Mechanism	Average Accuracy (%)	ΔParams (M)	ΔFLOPs (G)
SENet	96.42	0.016	0.011
CBAM	97.08	0.033	0.018
EMA	98.8	0.005	0.007

Note: ΔParams and ΔFLOPs denote the increase relative to the baseline model without attention mechanisms. Input size is (64, 8, 8).

**Table 8:** Accuracy (%) of different methods on cross-location (DE→FE) few-shot diagnosis task (10-way 5-shot).

Method	Accuracy (Average ± Standard Deviation)
SVM	46.91 ± 6.45
Reptile	85.34 ± 7.12
ProNet	88.72 ± 7.83
MAML	91.15 ± 5.71
GMAML	94.88 ± 4.24
Proposed method	97.63 ± 3.29

## Data Availability

The datasets analyzed during this study are available at https://engineering.case.edu/bearingdatacenter/download-data-file (accessed on 1 September 2025).
